# Medical Inventiveness in War: Some Minor Examples of Ingenuity

**Published:** 1915-02-13

**Authors:** 


### MEDICAL INVENTIVENESS IN WAR.

### Some Minor Examples of Ingenuity.

In the popular sense of the term a history of the war is a record of the operations of navies and armies, prefaced, perhaps, by a brief account of the diplomacy which failed to prevent the catastrophe. Even the more ambitious compilation, the materials for which are now being collected in some official bureau, will concern itself mainly with military movements and their significance and results. Both in the one case and the other it is with fights and fighters that the story is concerned. There are, however, many other aspects of the struggle which are worthy of attention, and indeed these are so numerous that no one mind may hope to note and weigh them all. The social, economic, and temperamental life of the whole world is more or less disturbed and modified, and consequences now hardly appreciable will in due course become manifest. One of the effects of the war is to stimulate ingenuity to devise new expedients to meet new situations, and some illustrations of this influence may be observed in the world of medicine. Relative to the large issues striving for settlement, such matters may appear trivial. They have, however, their importance, and it is well that due notice be taken of them.

One of the surgical problems now prominent—and for obvious reasons—is the detection, localisation, and removal of bullets and other foreign bodies which have entered the tissues. These necessities have created many activities in the $x$-ray departments of hospitals, and have led to definite advances. For the non-technical worker detailed descriptions of these advances would have little or no meaning, and we do not propose to attempt them. There are, however, two developments in the direction with which we are now concerned that seem worthy of record. One of these is an $x$-ray director, devised by Dr. Ironside Bruce. To the inexperienced it would seem a very simple matter to cut down upon and to remove a foreign body when this has once been localised by means of the fluorescent screen or by a skiagram. But those who have tried it know how easy it is to miss the way. The new director is intended to obviate this error. The patient is placed and indeed fixed in the position desired by the operator; the shadow of the foreign body is obtained on the exact centre of the fluorescent screen; and then, at once, the screen is replaced by a metal guide carrying a director, the point of which corresponds exactly to the centre of the screen. When this director is advanced through the tissues its point must inevitably impinge against the foreign body, the presence and general position of which have previously been made certain by an ordinary $x$-ray examination. To ensure success, great attention to detail is obviously necessary, but the general scheme of the proposal is of much interest, and its value has already been attested in practice. Another practical method we owe to Sir J. Mackenzie Davidson. Some years ago he interested himself in the application of the telephone to surgery, and under the modern demand he has extended and improved his method so that it is now possible to obtain an auditory signal from a metal body imbedded in the tissues. The surgeon is provided with a headpiece somewhat similar to those used by the clerks in a telephone exchange, while wires leading from this can be attached to any instrument—a probe, for example—with the certainty that as soon as contact is made with a metal body a signal of the fact will be conveyed to the surgeon's ear. The principle, as we have already said, is not new, but its most recent modification presents the apparatus in a very serviceable form.

Another surgical question of acute interest is the best method of treatment in the case of wounds, and especially of septic wounds. The virtues of such substances as carbolic acid, and iodine, and petrol have been energetically canvassed, and all of these, and others, have their advocates. What seems certain is that no general doctrine of universal application can be laid down, and that the surgeon must be guided by the peculiarities of the individual case. In this connection note ought to be taken of the results recorded by Mr. Herbert F. Waterhouse from the local application of ether to gunshot and other wounds. Of its value as an antiseptic he speaks in the highest terms, and it has the advantage of being almost painless. On the other hand, iodine, however useful as an agent for sterilising the skin, causes considerable pain when applied to wounds.

Minor medicine in the trenches is obviously a war development, and Dr. Tunnicliffe tells us that it may have to be practised apart from medical direction. In these circumstances a supply of medicines in easily measured doses is a necessity. The same claim arises in relation to some effective anodyne for the relief of pain in men wounded in the trenches and waiting the opportunity of removal to the rear. Here the patient may be more or less unconscious, and hence the question of medicines in a form easily swallowed has to be considered. Dr. Tunnicliffe suggests that all these conditions can be met by the incorporation of medicines with a suitable basis contained in a collapsible tube, and in such proportions that a given quantity of the paste or cream, say an inch, contains a suitable dose. In this fashion the difficulties of hypodermic medication, of tablets, cachets, etc., are avoided, and the medicine is delivered directly into the patient's mouth without the intervention of any vessel for measurement or administration. This and other new proposals show that the ingenuity of the profession is being stimulated by the conditions created by the war. Indeed, it is only necessary to read the correspondence columns, either of medical or of lay journals, to find that such happenings as frost-bite, the presence of pediculi, and other such evils are occasions for new methods of treatment or for the revival of forgotten methods.

